# Multilevel Context Learning with Large Language Models for Text-Attributed Graphs on Social Networks

**DOI:** 10.3390/e27030286

**Published:** 2025-03-10

**Authors:** Xiaokang Cai, Ruoyuan Gong, Hao Jiang

**Affiliations:** Electronic Information School, Wuhan University, Wuhan 430072, China; whuxkcai@whu.edu.cn (X.C.); 2020302121235@whu.edu.cn (R.G.)

**Keywords:** text-attributed graph, representation learning, large language models, graph neural networks

## Abstract

There are complex graph structures and rich textual information on social networks. Text provides important information for various tasks, while graph structures offer multilevel context for the semantics of the text. Contemporary researchers tend to represent these kinds of data by text-attributed graphs (TAGs). Most TAG-based representation learning methods focus on designing frameworks that convey graph structures to large language models (LLMs) to generate semantic embeddings for downstream graph neural networks (GNNs). However, these methods only provide text attributes for nodes, which fails to capture the multilevel context and leads to the loss of valuable information. To tackle this issue, we introduce the Multilevel Context Learner (MCL) model, which leverages multilevel context on social networks to enhance LLMs’ semantic embedding capabilities. We model the social network as a multilevel context textual-edge graph (MC-TEG), effectively capturing both graph structure and semantic relationships. Our MCL model leverages the reasoning capabilities of LLMs to generate semantic embeddings by integrating these multilevel contexts. The tailored bidirectional dynamic graph attention layers are introduced to further distinguish the weight information. Experimental evaluations on six real social network datasets show that the MCL model consistently outperforms all baseline models. Specifically, the MCL model achieves prediction accuracies of 77.98%, 77.63%, 74.61%, 76.40%, 72.89%, and 73.40%, with absolute improvements of 9.04%, 9.19%, 11.05%, 7.24%, 6.11%, and 9.87% over the next best models. These results demonstrate the effectiveness of the proposed MCL model.

## 1. Introduction

The rise in social networks has changed the way people acquire information, leading to a surge in online users [1]. For example, X (formerly known as Twitter) is one of the world’s largest social media platforms. Users can share short text messages in short posts commonly known as “tweets” (officially “posts”) and repost or comment other users’ content. Similarly, Sina Weibo is one of the most popular microblog platforms in China, similar to X but with some localized features. Users on such social platforms publish a large amount of text to express emotions, intentions, and opinions, and their interaction behaviors, such as reposting and commenting, create complex graph structures.

Text plays a crucial role in information propagation within the graph, while the graph structure provides abundant context for interpreting the text’s meaning. Therefore, analyzing text based on the graph structure can provide valuable information for social network tasks, such as user classification [2], personalized recommendations [3], and community detection [4].

Due to the rich textual information and complex graph structures present on social networks, the recent research topic in this field focuses on combining natural language processing (NLP) techniques and network science [5,6,7]. Contemporary researchers tend to represent these kinds of data on such social networks by text-attributed graphs (TAGs). A TAG is a graph where nodes or edges are associated with text, commonly found in domains like citation networks [8,9], web page hyperlink networks [10], and social networks [5]. Unlike traditional NLP methods that transform text attributes into shallow or hand-crafted features, such as bag-of-words [11] or skip-gram [12], the core of TAG representation learning lies in the integration of graph structure and textual information. Given that graph neural networks (GNNs) excel at capturing graph structures and large language models (LLMs) perform well in various natural language processing tasks, most existing TAG-based methods focus on frameworks that convey graph structures to LLMs in order to generate semantic embeddings for GNNs. For example, SimTeG [13] fine-tunes an LLM to generate semantic embeddings for a GNN through a consistent downstream task loss, while LMaaS [14] utilizes a pre-trained language model (PLM) as an interpreter which transforms the explainable texts generated by the LLM into embedding vectors for the GNN. DGTL [15] inputs the text information encoded by an LLM into a disentangled GNN to capture the graph structural information, then feeds the learned representation vectors into the LLM predictor.

**Figure 1:**
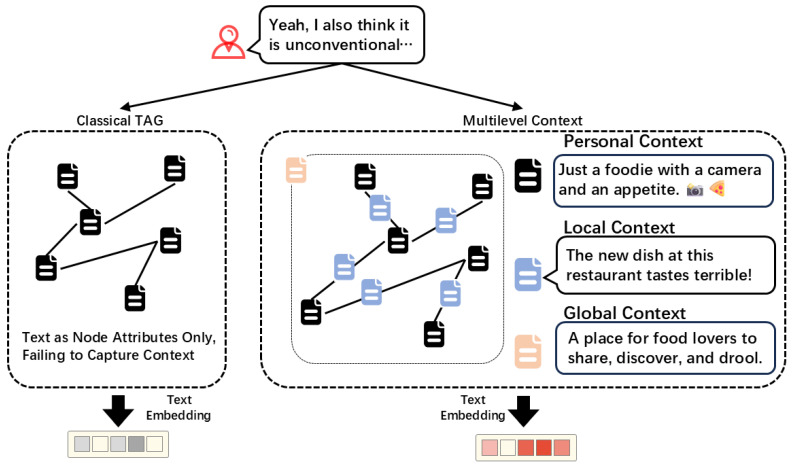
An example of someone describing something as “unconventional”. This could be interpreted as a positive comment without context. The personal context shows that the user is a “foodie”. The local context indicates that the person he is replying to or commenting on has just expressed dissatisfaction with a certain food. The global context suggests that he is participating in a discussion about food. Based on the above multilevel context, this text is more likely to be interpreted as a criticism.

However, these methods only provide text attributes for nodes, which fail to capture the multilevel context on social networks and lead to the loss of valuable information from the outset. The neglected context is crucial in the analysis of users’ posts. First, online accounts typically include supplementary personal descriptions, such as occupation, hobbies, and education, which are essential for understanding their posts. Meanwhile, interactions such as reposts and comments provide important local context to clarify the meaning of individual texts. Furthermore, users’ current topics offer significant global context, as the same expression may have different meanings in different topics. As shown in Figure 1, when a user describes something as “unconventional” on the social network, it could have opposite meanings depending on the context. If it is used to describe a piece of art, it could express admiration. If used to describe food, it is more likely to be a subtle criticism. The different levels of context mentioned above, i.e., the personal context, the local context, and the global context, can provide important information to clarify the meaning. Therefore, it is essential to capture and utilize the multilevel context for semantic embeddings of users’ posts.

To capture the complex multilevel context and generate semantic embeddings for downstream tasks, the following challenges must be addressed: (1) How to model both multilevel context and graph structures in TAGs.

TAGs that only provide text attributes for nodes fail to capture multilevel contexts, which in turn affects the quality of the semantic embeddings. Firstly, providing only text attributes for user nodes fails to differentiate between personal descriptions and posts. Additionally, interactions such as reposts and comments between users form text pairs, but classical TAGs fail to capture the semantic relationships based on these text pairs. For example, when two users engage in interactions based on different text pairs, classical TAGs struggle to distinguish between them. Therefore, it is essential to design TAGs that capture more detailed information, enabling the simultaneous modeling of multilevel context and graph structure. (2) How to leverage the multilevel context to generate semantic embeddings for downstream tasks. Enabling LLMs to leverage multilevel context is also challenging because the multilevel context on social networks is distinguished not only in terms of granularity but also in form. The personal context is reflected at the node level and may vary significantly in form across different users. The local context is represented through the edges, and the complex semantic relationships are difficult to describe using natural language. Additionally, the global context is embodied throughout the overall graph and does not have predefined descriptions. Therefore, directly applying the multilevel context as prompts for LLMs is not practical. This highlights the importance of developing a more effective approach to utilize the multilevel context based on the designed TAG.

To solve these challenges, we propose the Multilevel Context Learner (MCL) model, which can leverage multilevel context on social networks to enhance the semantic embedding capabilities of LLMs for downstream tasks. First, we model the social network as a multilevel context textual-edge graph (MC-TEG), with personal descriptions as node attributes and interaction texts as edge attributes. Node attributes capture personal context, while edge attributes provide the basis for utilizing local context. Second, the proposed MCL leverages LLMs’ reasoning abilities to infer and update global context from posted text. Then, it combines the local context through relevant edges with personal and global context to generate semantic embeddings for each node. Moreover, a group of tailored bidirectional dynamic graph attention network (GAT) layers [16] are developed to further distinguish the weight information on social networks. Two types of attention are trained separately to collectively represent the relationships among nodes. To demonstrate the effectiveness of our proposed model, we evaluated it on the fundamental graph representation learning task: node classification. Extensive experiments on real social network datasets demonstrate the effectiveness of MCL.

Our main contributions are summarized as follows:

- We model the social network as a multilevel context textual-edge graph (MC-TEG). Personal descriptions are regarded as node attributes, while interaction texts are treated as edge attributes, effectively capturing both graph structure and semantic relationships.
- We propose the Multilevel Context Learner (MCL) model, which utilizes LLMs’ reasoning abilities to leverage multilevel context for generating semantic embeddings. The proposed bidirectional dynamic graph attention layers further distinguish the weight information.
- Experimental evaluations on six social network datasets demonstrate the effectiveness of the proposed MCL model, which consistently outperforms all baseline methods across all datasets.

## 2. Related Works

In this section, we review related works on TAG representation learning and compare the existing approaches with the proposed MCL model on social networks.

### 2.1. Text-Attributed Graphs

TAGs are employed to represent structured data where nodes or edges are associated with text attributes [17], which are ubiquitous across various domains, including citation networks [8,9], web page hyperlink networks [10], and social networks [5]. TAGs have attracted considerable attention in the field of graph machine learning in recent years [13,18,19]. GNNs have been proven to be an effective framework for graph machine learning following the neighborhood aggregation scheme [20,21]. Classical TAG representation learning methods based on GNNs convert text attributes into shallow or hand-crafted features such as bag-of-words [11] or skip-gram [12] representations. The bag-of-words model represents text by counting the occurrences of each word within a document, disregarding grammar and order. The skip-gram model focuses on predicting the surrounding words within a fixed window to capture semantics. However, these methods are unable to capture polysemy and the semantic relationships between words, resulting in only basic semantic embeddings.

With the development of natural language processing (NLP) technologies, pre-trained language models (PLMs) [22] and topic models (TMs) [23,24] are used to handle text attributes, which can learn contextualized language representations and document embeddings. PLMs achieve deeper semantic understanding by first being trained on large-scale datasets and then fine-tuning for specific tasks. For example, TextGNN [25] extends the twin tower structured encoders of PLM with complementary graph information from user historical behaviors to generate better text representations. AdsGNN [26] leverages PLMs to obtain text representations at the granularities of nodes, edges, and tokens, respectively. In contrast to PLMs, TMs assume that texts contain a small number of latent topics that summarize distinct and broad concepts. Adjacent-Encoder [27] and DBN [28] use latent topics to generate the textual content of a document’s neighbors based on GNNs. Although PLM-based models and TM-based models can achieve deeper semantic embeddings, the encoding process remains independent of structural information and therefore does not fully leverage the complex semantic relationships in TAGs. Consequently, there is a growing need for the integration of text attributes and graph structure for TAG representation learning.

### 2.2. LLMs for TAGs

With the remarkable capabilities demonstrated by LLMs in various NLP tasks, recent research studies have explored the use of LLMs to address graph-related tasks. By treating graph problems as conventional NLP problems, pioneer researchers convert graph data into a representation that is comprehensible to LLMs on synthetic graph tasks. NLGraph [29] treats graphs as natural language descriptions and evaluates the performance of LLMs on various graph reasoning tasks, including connectivity, shortest path, maximum flow, and the simulation of GNNs. GPT4Graph [30] also converts graphs into specific vectors to evaluate the capabilities of LLMs in structural and semantic understanding tasks. InstructGLM [31] enhances the vocabulary by introducing new tokens for each node in the TAG, allowing LLMs to be fine-tuned for handling various TAG tasks in a generative way.

More recently, there has been a growing exploration into applying LLMs to TAGs. SimTeG [13] fine-tunes the LLM to generate semantic embeddings for the GNN through the consistent downstream loss function such as link prediction or node classification. LMaaS [14] utilizes a PLM model as the interpreter, converting the explanation and prediction from LLMs into embedding vectors for GNNs. DGTL [15] inputs the text information encoded by the LLM into disentangled GNNs to capture the structural information, and then feeds the learned representation vectors into the LLM predictor.

However, most existing representation learning approaches on TAGs focus on designing frameworks to convey graph structures to LLMs while overlooking the graph modeling process. When applied to social networks, traditional TAG structures fail to accurately capture the heterogeneous texts and the structural information. As a result, although these methods have designed sophisticated frameworks to enable LLMs to leverage graph structures, a significant amount of valuable information is lost in modeling the graph structures, which leads to limitations in fully exploiting the potential power of LLMs. In our method, we propose the LLM Enhanced Text Learner on Social Networks (MCL) model, which models the social network as a multilevel context textual-edge graph (MC-TEG). MCL captures and captures both graph structure and semantic relationships on social networks to enhance the reasoning capabilities of LLMs for downstream tasks.

## 3. Problem Formulation and Preliminaries

In this section, we introduce notation and formalize some concepts related to textual-edge graphs, graph neural networks, and large language models.

### 3.1. Textual-Edge Graphs

A textual-edge graph can be formulated as G=(V,E,y), where V denotes the set of nodes, E∈V×V denotes the set of edges, and **y** denotes the the labels of nodes. In a TEG, each node v∈V is associated with a text description, and each edge $e_{ij}\in E$ also contains its text description, which is absent in traditional TAGs. These textual descriptions provide rich contextual information about the complex relationships between nodes, enabling a more detailed and comprehensive representation of data relations than traditional TAGs.

In this paper, we focus on node classification, one of the most typical tasks on graphs. We adopt the semi-supervised settings, where all the text information and the adjacency matrix A are given during the training procedure, while only a part of the node labels $\left\{y_{u}|u\in V_{tr}\right\}$ are provided, where $V_{tr}$ is the training node set. The task aims at predicting the labels of $\left\{y_{u}|u\in V_{te}\right\}$, where $V_{te}$ is the set of test nodes.

### 3.2. Large Language Models

LLMs have introduced a new paradigm for task adaptation known as “pre-train, prompt, and predict”. In this paradigm, the LLM is first pre-trained on a large corpus of text data to learn general language representations. Instead of fine-tuning the model, a natural language prompt that defines the task and context is then provided to the model. The prompt can be presented in various formats, ranging from a concise sentence to a more extensive passage, and may incorporate supplementary details or constraints to direct the model’s behavior accordingly. Based on the prompt and input tokens, the model generates the output directly. Formally, for the sequence of input tokens $x=(x_{1},x_{2},\ldots ,x_{q})$ and the prompt prom, we can concatenate them into a new sequence $\hat{x}=\left(prom,x_{1},x_{2},\ldots ,x_{q}\right)$. Then, the probability of the output sequence $s=(s_{1},s_{2},\ldots ,s_{m})$ given $\hat{x}$ is

(1) $$p\left(s|\hat{x}\right)=\prod_{i=1}^{m}p\left(s_{i}|s_{<i},\hat{x}\right)$$

where $s_{<i}$ represents the prefix of sequence *s* up to position i−1, and $p(s_{i}|s_{<i},\hat{x})$ represents the probability of generating token $s_{i}$ given $s_{<i}$ and $\hat{x}$.

### 3.3. Graph Neural Networks

Graph neural networks are a class of deep learning models specifically designed to handle graph-structured data [32]. GNNs extend the capabilities of traditional neural networks, enabling direct operation on graph structures, thereby capturing complex relationships and dependencies between nodes. GNNs typically follow a message-passing scheme where nodes aggregate information from their neighbors in each layer, formulated as

(2) $${m_{i}}^{\left(l\right)}=\mathrm{Agg}\left({h_{j}}^{\left(l\right)}|j\in N_{i}\right)$$

(3) $${h_{i}}^{\left(l+1\right)}=\mathrm{Update}\left({h_{i}}^{\left(l\right)},{m_{i}}^{\left(l\right)}\right)$$

where ${h_{i}}^{\left(l\right)}$ is the representation vector of node *i* at the *l*-th layer, $N_{i}$ is the neighbors of node *i*, **Agg(·)** is the aggregation function, and **Update(·)** is an updating function that typically includes linear functions and activation functions. For node classification, the output **ŷ** of GNNs is a normalized vector, where the dimension corresponds to the number of node categories and the values represent the probabilities of the node belonging to the corresponding category.

## 4. Method

**Figure 2:**
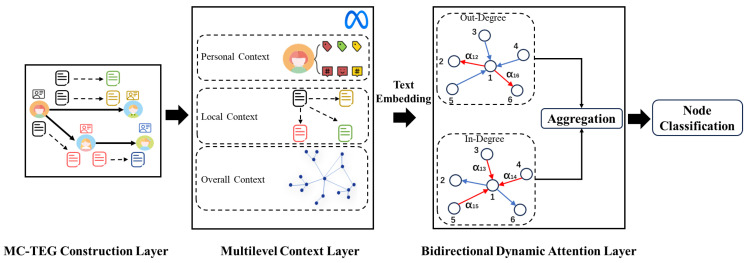
An overview of our proposed Multilevel Context Learner (MCL) model. First, we model the social network as a multilevel context textual-edge graph (MC-TEG). Second, we leverage the large language model (LLM) to capture multilevel contexts and generate embeddings for downstream tasks. Third, we train the bidirectional dynamic attention layer for node classification.

In this section, we describe our proposed MCL model for node classification on social networks. An overall framework of our method is shown in Figure 2; it involves three main steps: (1) constructing an MC-TEG based on social networks; (2) utilizing LLMs to extract multilevel contexts and generate embeddings; and (3) training a bidirectional dynamic graph attention for prediction.

### 4.1. MC-TEG Construction Based on Social Networks

The first step in our method was to construct an MC-TEG that captures both graph structure and semantic relationships based on social network data. We collected textual data from real social networks based on tags and keywords and obtained personal descriptions of corresponding accounts. The dataset may include considerable noise due to users attaching irrelevant tags to their posts in an effort to increase visibility and engagement. Additionally, some keywords may appear in discussions across multiple topics and could carry different meanings. Given the scale of the dataset, manually filtering out this noise is not feasible, which highlights the necessity of using LLMs to leverage multilevel context to obtain accurate semantic embeddings.

We treat users as nodes in the MC-TEG, with personal descriptions serving as the text attributes $t_{i}$ for node *i*. The interaction texts between node *i* and node *j* are represented as pairs of original and repost or comment texts, serving as the corresponding edge attributes $t_{ij}$. Notably, $t_{ij}$ and $t_{ji}$ are distinct, as the edges are directed. Moreover, if there are multiple interactions between users, the corresponding $t_{ij}$ will include multiple text pairs.

**Figure 3:**
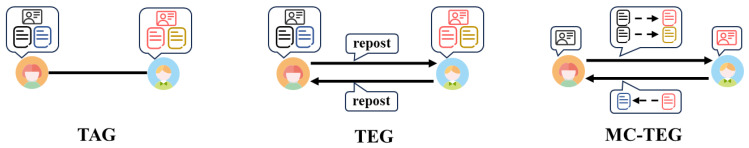
A simple comparison of our MC-TEG with other graphs. Traditional TAG treats all text as node attributes, making it unable to handle interactions or distinguish between user descriptions and post texts. Although TEG adds textual attributes to edges, it only reflects the direction of interactions and cannot handle multiple interactions. Our MC-TEG not only distinguishes between user descriptions and post texts but also further differentiates the relationships and directions of interactions.

As shown in Figure 3, our MC-TEG provides textual attributes for both nodes and edges. The user descriptions serve as the textual attributes of the nodes, while the textual pairs representing interactions between users act as the textual attributes of the edges. The textual pairs on the edges not only effectively handle multiple interactions, but also distinguish the directionality of these interactions. Additionally, this structure facilitates the utilization of personal contexts through textual attributes on nodes and enhances the updating and utilization of global contexts.

In summary, the constructed MC-TEG preserves the graph structure while providing a foundation for LLMs to leverage multilevel context.

### 4.2. LLM-Based Multilevel Context Extraction and Embedding Generation

In this subsection, we utilize the powerful reasoning and comprehension abilities of LLMs to leverage personal context, local context, and global context, and then generate semantic embeddings based on the multilevel context for downstream GNNs.

#### 4.2.1. Personal Context

The challenge in extracting user context lies in the fact that personal descriptions are unstructured. There are differences in style and content across personal descriptions of different users, making it difficult to unify them. This issue can be effectively addressed by designing specific prompts for the LLM. We design a fixed template and set key information as tokens that the LLM needs to predict based on personal descriptions. At the same time, we determined a set of default values in the prompt to handle cases where some personal descriptions may lack the key information. This structured output standardizes the format of the personal context for each node, facilitating subsequent utilization. The personal context of node *i* is

(4) $$c_{i}=f_{\mathrm{pr}}\left(t_{i}\right)$$

where $c_{i}$ is structured personal context of node *i*, and $f_{\mathrm{pr}}$ is the LLM prediction function for personal context.

#### 4.2.2. Local Context

We treat the 1-hop interaction texts among users as the local context. This is reasonable because it reflects the most direct semantic relationships, and increasing the number of hops would lead to an exponential increase in complexity. In the designed MC-TEG, interaction texts are already treated as edge attributes, which effectively reflect the local context. At the same time, the directionality of the edges also introduces distinctions in the local context. We denote $r_{in}$ and $r_{out}$ as the local contexts when node *i* acts as the target node and the source node, respectively, which is reflected in the differing positions of node *i*’s text within the text pairs $t_{ij}$ or $t_{ji}$.

#### 4.2.3. Global Context

Online users often engage in discussions based on specific topics, which provides valuable global context. However, these topics are not explicitly predefined and are accompanied by significant noise. We fully utilize the powerful reasoning capabilities of LLMs by constructing prompts from the text pairs on the edges to infer the topics within the graph. Since the input consists of text pairs, some important texts may be repeatedly included. LLMs can reduce the impact of noise in the topic-update process by effectively leveraging patterns identified in repeated texts. The global context is inferred as follows:

(5) $$T=f_{\mathrm{gl}}\left(\underset{e_{ij}\in E}{⋃}t_{ij}\right)$$

where T is the inferred global context, $f_{\mathrm{gl}}$ is the LLM-specific function for the global context, ⋃ represents the iterating progress, and $t_{ij}$ is the text pair associated with edge $e_{ij}$.

#### 4.2.4. Semantic Embedding

**Figure 4:**
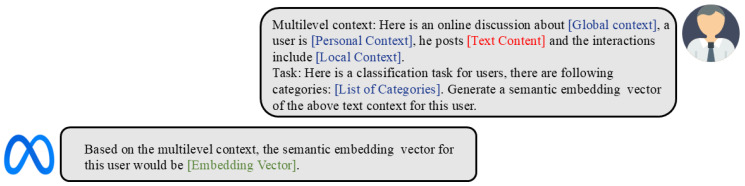
The illustration of prompts and typical responses. The blue parts indicate the texts populated based on the content of the dataset, including the multilevel context and the list of categories. The red parts indicate the text for semantic embedding. The green parts indicate the output given by the LLM.

To fully leverage the LLM’s capability to understand and model complex patterns and semantics in MC-TEG, we injected the associated multilevel context into the LLM, which includes $c_{i}$ (personal context), $r_{in}$ and $r_{out}$ (local context), and T (global context). Each context level provides valuable information that helps the LLM form a comprehensive understanding of the node’s semantics within the MC-TEG. Specifically, we reserved a set of token positions for placing the multilevel context in the prompt input. Even if the different levels of context are not unified in form, the form of our input will still allow the LLM to think that they are aligned with the natural semantic space that humans can understand, as shown in Figure 4. Through this approach, we enabled the LLM to benefit from a comprehensive understanding of both the graph structure and textual information, generating semantic embeddings for downstream GNNs tasks. These semantic embeddings facilitate a direct gradient flow to the GNNs, resulting in more accurate and informative gradient updates. This fusion of language modeling and graph representation learning enables our MCL model to leverage the multilevel context captured by the LLM alongside the structural patterns learned by the GNNs, driving effective learning and enhanced performance.

### 4.3. Prediction by Bidirectional Dynamic Graph Attention Layers

As a classic GNN, the graph convolution mechanism uses a uniform weight matrix to aggregate features, which is unsuitable for social networks because different neighbors have distinctly varying impacts on different users. The attention mechanism can effectively address this issue, which is initially employed in computer vision [33] and then in NLP [22]. The attention mechanism has subsequently been proven to be competitive in graph analysis and has led to the popularity of graph attention networks [34]. An attention score is denoted as $\alpha_{ij}$, which indicates the importance from the neighbor *j* to the node *i*. The unnormalized attention score for edge (i,j) in layer *m* is computed as follows:

(6) $$e\left({h_{i}}^{\left(m\right)},{h_{j}}^{\left(m\right)}\right)=\mathrm{LeakyReLU}\left(a^{T}\cdot \left[W{h_{i}}^{\left(m\right)}∥W{h_{j}}^{\left(m\right)}\right]\right)$$

where $a\in R^{2d_{m+1}}$ and $W\in R^{d_{m+1}\times d_{m}}$ are learned in the training process, and ∥ represents vector concatenation. After computing all $e\left({h_{i}}^{\left(m\right)},{h_{j}}^{\left(m\right)}\right),j\in N_{i}$, a softmax layer is used to normalize them and obtain the attention score $\alpha_{ij}$:

(7) $$\alpha_{ij}=\frac{\exp\{e\left({h_{i}}^{\left(m\right)},{h_{j}}^{\left(m\right)}\right)\}}{\sum_{j^{\prime }\in N_{i}}\exp\left\{e\left({h_{i}}^{\left(m\right)},{h_{j^{\prime }}}^{\left(m\right)}\right)\right\}}$$

Then, the attention weighted average in $N_{i}$ is used to update the representation of node *i* in layer m+1:

(8) $${h_{i}}^{\left(m+1\right)}=\sigma \left(\sum_{j\in N_{i}}\alpha_{ij}\cdot W{h_{j}}^{\left(m\right)}\right)$$

where σ is a nonlinear function. Although this attention mechanism can distinguish the weight matrix, it still has a limitation: nodes are ranked relatively equally for nodes, only differing in absolute values. This significant limitation diverges from the nature of social networks because the importance ranking of different users varies. To address this limitation, we use the dynamic attention [16], where the order of operations in the scoring function is modified as follows:

(9) $$\begin{array}{c} e\left({h_{i}}^{\left(m\right)},{h_{j}}^{\left(m\right)}\right)=a^{T}\mathrm{LeakyReLU}\left(W\cdot [{h_{i}}^{\left(m\right)}∥{h_{j}}^{\left(m\right)}]\right) \end{array}$$

where the simple modification makes a significant difference in the expressiveness of the attention function.

However, simply using dynamic attention is still not adequate to simulate the characteristics of social networks. In Equation (10), the attention scores are normalized among all neighbors of the node without distinguishing between out-degree and in-degree. But on social networks, retweeting and being retweeted not only represent the direction of edges but also reflect the active and passive nature of user behavior, thus requiring attention to be distinguished accordingly. Therefore, we propose bidirectional dynamic graph attention layers. The out-degree attention and in-degree attention are trained with distinctions during the normalization process as follows:

(10) $$\begin{array}{cc} e^{\mathrm{Out}}\left(h_{i},h_{j}\right) & =a_{1}^{T}\mathrm{LeakyReLU}\left(W_{1}\cdot \left[h_{i}∥h_{j}\right]\right) \end{array}$$

(11) $$\begin{array}{cc} e^{\mathrm{In}}\left(h_{i},h_{j}\right) & =a_{2}^{T}\mathrm{LeakyReLU}\left(W_{2}\cdot \left[h_{i}∥h_{j}\right]\right) \end{array}$$

(12) $$\begin{array}{cc} {\alpha_{ij}}^{\mathrm{Out}} & =\frac{\exp\left\{e^{\mathrm{Out}}\left(h_{i},h_{j}\right)\right\}}{\sum_{j^{\prime }\in N_{i}^{\mathrm{Out}}}\exp\left\{e^{\mathrm{Out}}\left(h_{i},h_{j^{\prime }}\right)\right\}} \end{array}$$

(13) $$\begin{array}{cc} {\alpha_{ij}}^{\mathrm{In}} & =\frac{\exp\left\{e^{\mathrm{In}}\left(h_{i},h_{j}\right)\right\}}{\sum_{j^{\prime }\in N_{i}^{\mathrm{In}}}\exp\left\{e^{\mathrm{In}}\left(h_{i},h_{j^{\prime }}\right)\right\}} \end{array}$$

where ${N_{i}}^{Out}$ and ${N_{i}}^{In}$ represent the out-degree neighbors and in-degree neighbors, respectively. $a_{1}$, $a_{2}$, $W_{1}$, and $W_{2}$ denote different training parameters while $h_{i}$ is shared between the out-degree layer and in-degree layer. The representation vector will be updated to the weighted sum of two types of attention as follows:

(14) $$\begin{array}{c} \begin{array}{c} {h_{i}}^{\left(m+1\right)}=\sigma \left(\lambda \sum_{j\in N_{i}^{\mathrm{Out}}}{\alpha_{ij}}^{\mathrm{Out}}\cdot W_{1}{h_{j}}^{\left(m\right)}+\left(1-\lambda \right)\sum_{j\in N_{i}^{\mathrm{In}}}{\alpha_{ij}}^{\mathrm{In}}\cdot W_{2}{h_{j}}^{\left(m\right)}\right) \end{array} \end{array}$$

where λ is the hyperparameter. ${h_{i}}^{\left(m+1\right)}$ is shared in the next layer and the node representation is iteratively updated according to Equations (10)–(14). Due to the presence of multiple categories, we use the cross-entropy loss function:

(15) $$L=-\frac{1}{|V_{tr}|}\sum_{v\in V_{tr}}\sum_{j=1}^{K}y_{vj}\log\left({\hat{y}}_{vj}\right)$$

where $V_{tr}$ is the training node set, $|V_{tr}|$ denotes the size of this space, *K* is the number of categories, $y_{vj}$ is the true label vector element for node *v* and category *j*, and ${\hat{y}}_{vj}$ is the predicted probability of the node *v* belonging to the category *j*. To prevent overfitting, we add a regularization term. The final loss function is as follows:

(16) $$L_{total}=L+\sum_{i=1}^{2}\theta_{i}{∥W_{i}∥}_{2}^{2}$$

where $∥W_{i}{∥}_{2}^{2}$ is the L2 norm of each weight matrix $W_{i}$, and $\theta_{i}$ is the corresponding coefficient.

## 5. Experiment

In this section, we will compare the MCL model with several baseline methods and demonstrate the effectiveness of node classification on social networks. The experiment was conducted on a server with an Ubuntu 22.04 LTS system.(Canonical Ltd., London, United Kingdom). The server has 96 cores and a clock speed of 2.5 GHz. The GPU used by the MCL model is Nvidia GeForce RTX 4090. (NVIDIA Corporation, Santa Clara, CA, USA).

### 5.1. Datasets

To verify the validity and robustness of our model, we collected data related to political elections from X (formerly Twitter) and data related to school food safety from Sina Weibo. X is one of the world’s largest social media platforms, while Sina Weibo is one of the most popular microblog platforms in China, similar to X but with localized features. Users on such social media platforms can add words or phrases starting with “#” (also known as hashtags) to categorize their posts and make them discoverable to a wider audience. To collect the data required for this study, we utilized publicly available APIs provided by the social media platforms (https://developer.twitter.com (accessed on 10 January 2024) and https://open.weibo.com (accessed on 3 June 2023)). These APIs enabled us to systematically retrieve relevant data, including posts, comments, and metadata, based on specific hashtags, all in a structured and compliant manner. All data were carefully de-identified to ensure that no personal information was leaked. Subsequently, these data underwent preprocessing, which included handling missing values and excluding posts in languages other than the target language. We constructed the network structure based on symbols in the text that reflect reposting and replying relationships, such as [rtt] and ‘@’. The detailed description of the datasets is as follows:

- Datasets on X. These three English datasets record discussions on three topics related to the 2024 United States Presidential Election on X. They include information about the posting users, the content of the posts, posting times, retweet counts, and content relationships. The recording period covers discussions from 10th January 2024 to 10th February 2024. Users in these datasets are categorized into three classes: supporters of Trump, neutral users, and supporters of Harris. The three datasets are arranged in ascending order of size as X_A, X_B, and X_C. X_A contains data related to the hashtags #DemocraticParty and #RepublicanParty, X_B contains data related to #Harris and #Trump, and **X_C** contains data related to #2024UnitedStatesElections.
- Datasets on Sina Weibo. The three Chinese datasets record discussions on three topics related to school food safety on Sina Weibo. They also include information about the posting users, the content of the posts, posting times, retweet counts, and content relationships. The recording period spans 3rd June 2023 to 30th June 2023. Users in these datasets are categorized into three classes based on their level of support for students: supporters, neutral users, and opponents. The three datasets are arranged in ascending order of size as Weibo_A, Weibo_B, and Weibo_C. Weibo_A contains data related to hashtags about media coverage, Weibo_B contains data related to hashtags about school statements, and Weibo_C contains data related to hashtags about official statements.

The statistics of all datasets are shown in Table 1.

### 5.2. Experimental Setup

In the proposed MCL model, we used GPT-4 as the LLM to generate semantic embeddings. We compared our model with baselines from the following two categories:

- GNN Predictors. We considered different GNN-based models to enhance the node features on TAG. Our baselines include mT5 [35] and DeBERTa [36]. We selected the most suitable GNN backbones based on the descriptions of their methods.
- LLM Predictors. We also considered using different LLMs as baselines, where the text is directly input into the models for prediction. We performed predictions using Llama [37], ChatGLM [38], QwenLM [39], and ERNIE BOT [40].

For all methods, we adopted the classical semi-supervised learning setting, randomly selecting 10% of the data from each category to form the training set. We directly used classification accuracy as the evaluation metric.

### 5.3. Overall Evaluation

The results of our comparison with the baselines are shown in Table 2. From the table, we can find that LLM predictors are generally better GNN predictors, indicating that the text on social networks cannot be adequately represented by solely pre-trained models. The reasoning ability of LLMs allows them to adapt and better understand these complexities. Our MCL model outperforms all baselines on all six datasets, demonstrating its effectiveness in both Chinese and English datasets. Unlike LLM-based predictors, our approach leverages the multilevel context on social networks, which is crucial for obtaining accurate text embeddings and enhancing the comprehensibility of the model’s decision-making process. In addition, we propose tailored bidirectional dynamic graph attention layers to further distinguish the weight information among nodes, which aligns more closely with the structural characteristics of social networks. Our MCL model excels by fully leveraging multilevel context and graph structure within social networks. As the dataset expands, its performance remains robust and relatively stable, whereas baselines typically encounter a decline in effectiveness.

### 5.4. Ablation Study

In this section, we designed ablation experiments to separately analyze the contributions of different levels of context and the bidirectional dynamic attention to the final results.

#### 5.4.1. The Multilevel Context Layers

**Figure 5:**
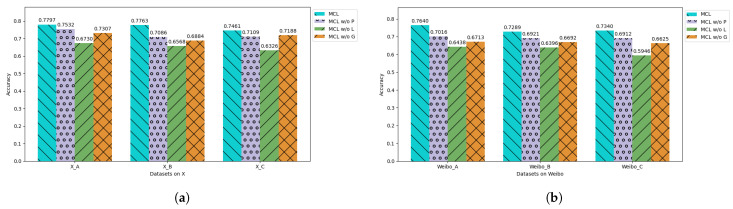
The results of the ablation experiment on different levels of context. MCL w/o P, MCL w/o L, and MCL w/o G, respectively, represent models where the personal context, local context, and global context are removed from the prompt. Subfigure (**a**) shows the ablation results of datasets on X, while subfigure (**b**) shows the ablation results of datasets on Sina Weibo.

To analyze the impact of different contexts on semantic embeddings, we conducted ablation experiments by removing token positions for personal, local, and global context in the input. As shown in Figure 5, removing context at any level affects the final results. The removal of local context has the largest impact, as user interactions in social networks are closely tied to text semantics. In contrast, removing personal context has the least impact, which we attribute to the fact that most user descriptions are either irrelevant or missing, so the personal context is set to default values, resulting in minimal impact.

#### 5.4.2. The Bidirectional Dynamic Attention Layers

**Figure 6:**
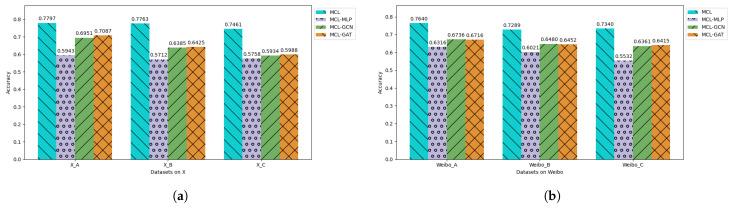
The results of the ablation experiment on bidirectional dynamic attention layer. The bidirectional dynamic attention layer is, respectively, replaced by multilayer perceptron (MLP), graph convolutional network (GCN), and graph attention network (GAT) layers. Subfigure (**a**) shows the ablation results of datasets on X, while subfigure (**b**) shows the ablation results of datasets on Sina Weibo.

We further analyzed the effect of the GNN backbone in the MCL model by replacing the bidirectional dynamic attention layer with multilayer perceptron (MLP), graph convolutional network (GCN), and GAT layers, respectively. The results of the ablation experiment are shown in Figure 6.

Our observations are as follows: (1) Our MCL model demonstrates the best performance. This indicates that the bidirectional dynamic attention layer indeed captures the structural information and is better suited for the directed nature of social networks. (2) The MLP backbone performs the worst. This is to be expected, as it cannot model graph-based dependencies and relationships, limiting its capacity to capture the complex structure of social networks. (3) The performance of the GAT backbone and GCN backbone is moderate. Although they leverage structural information, they do not distinguish the importance ranking of neighboring nodes, which diverges from the nature of social networks. (4) As the dataset size increases and the structure becomes more complex, the performance of our MCL model is not significantly affected, while the performance of the GAT and GCN architectures tends to deteriorate.

**Figure 7:**
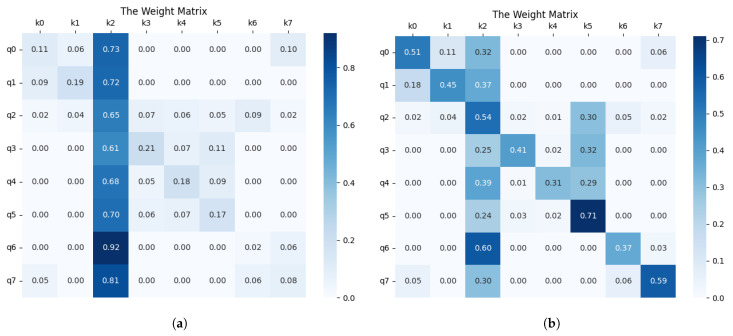
A comparison of the attention weight metrics, where $\left(q_{i},k_{j}\right)$ represents the attention of node *i* to node *j*. Subfigure (**a**) shows the attention weight matrix of classical GAT layers, while subfigure (**b**) shows the attention weight matrix of our bidirectional dynamic attention layer.

To quantify the contribution of our bidirectional dynamic graph attention layer, we sampled a network containing eight nodes and compared the weight matrices of the traditional GAT layer with those of our bidirectional dynamic attention. As shown in Figure 7, the traditional GAT assigns the highest weight to the second node for all nodes, indicating that the representation of each node is overly influenced by the second node. This does not capture the diversity of user attention in social networks. In contrast, our bidirectional dynamic attention layer produces unique weight rankings for each node, highlighting its effectiveness in capturing the complex structure of social networks.

## 6. Discussion

In the present work, we propose the Multilevel Context Learner (MCL) model to tackle the challenge of text-attributed graph (TAG) representation learning on social networks. Specifically, we model the social network as a multilevel context textual-edge graph (MC-TEG), effectively capturing both graph structure and multilevel context. Our method enables the large language models (LLMs) to leverage multilevel context to generate semantic embeddings for downstream tasks. The tailored bidirectional dynamic graph attention layers further capture the complex structural relationships of social networks. The impressive experimental results strongly demonstrated the effectiveness of MCL.

On the other hand, our method provides a new perspective on TAG representation learning. It emphasizes the importance of modeling TAGs for specific scenarios, which can capture valuable information for downstream tasks. Based on the constructed TAG structure, it is reasonable and effective to design models that leverage LLMs and GNNs. In future work, we will explore additional TAG structures to improve the effectiveness of TAG representation learning for other scenarios. For example, a hierarchical text-attributed graph is more suitable for capturing a hierarchical graph structure in citation networks, while a heterogeneous text-attributed graph is better suited for networks with different types of nodes, such as product recommendations or movie reviews. Furthermore, we will further explore the adaptation of MCL to other types of networks, such as biological networks, or power grids in Appendix A.

## Figures and Tables

**Table 1:** Dataset statistics.

Platform	Dataset	Nodes	Edges	Classes
X	X_A	1394	1297	3
	X_B	2750	3242	
	X_C	4432	6484	
Sina Weibo	Weibo_A	996	878	3
	Weibo_B	2168	2172	
	Weibo_C	3867	4344	

**Table 2:** Performance comparison with baselines.

	X			Sina Weibo		
Dataset	X_A	X_B	X_C	Weibo_A	Weibo_B	Weibo_C
mT5	0.5646	0.5684	0.5560	0.5682	0.5585	0.5508
DeBERTa	0.5854	0.5953	0.5776	0.5391	0.5544	0.6020
QwenLM	0.6714	0.6578	0.5952	0.6164	0.6305	0.5702
ERNIE	0.6399	0.6458	0.6173	0.6084	0.6019	0.5833
ChatGLM	0.6851	0.6796	0.6356	0.6345	0.6526	0.6214
Llama	0.6894	0.6844	0.6293	0.6736	0.6678	0.6353
MCL	0.7798	0.7763	0.7461	0.7640	0.7289	0.7340

## Data Availability

Data for this article can be obtained by contacting the corresponding author.

## Appendix A. Additional Experiments

To validate the performance of the MCL model on different networks, we conducted experiments on Protein–Protein Interaction (PPI) networks. We collected the network related to the nagA-2 protein of Rhodopirellula baltica from the STRING database. The dataset includes 61 nodes and 498 edges, where nodes represent proteins and edges represent various types of interactions (with 8 distinct edge types). Nodes are classified into six groups based on their functions and metabolic pathways. We adapted the construction of MC-TEGs to fit this network’s characteristics. Specifically, we used protein domains and descriptions as node features, and protein interaction types as edge features to differentiate interactions between the same nodes. We then conducted the node classification task under the same experimental setup, as shown in Table A1. The GNN predictors performed poorly due to their inability to handle the specialized terminology of protein domains. While LLM predictors can understand these terms, they cannot leverage the complex network structure. Our MCL model, however, captures both the specialized terms and the intricate context of protein interactions, delivering the best performance. These results demonstrate the generalization ability of the MCL model.

**Table A1:** The results on PPI dataset.

Methods	Accuracy
mT5	0.3061
DeBERTa	0.3673
QwenLM	0.5235
ERNIE	0.5102
ChatGLM	0.5306
Llama	0.5714
MCL	0.6122
